# Effects of Rehabilitation and Hospital Art on Mood of Inpatients in a Rehabilitation Ward: A Preliminary Study

**DOI:** 10.7759/cureus.87280

**Published:** 2025-07-04

**Authors:** Hiroo Koshisaki, Makoto Oura, Daishi Ogawa, Sachiko Nakai, Kanako Nojiri, Yukihiro Shimizu

**Affiliations:** 1 Department of Rehabilitation, Nanto Municipal Hospital, Nanto, JPN; 2 Department of Internal Medicine, Nanto Municipal Hospital, Nanto, JPN

**Keywords:** activities of daily living, hospital art, mood, older adults, rehabilitation

## Abstract

Introduction: Improvements in activities of daily living are closely linked to patients’ moods. While hospital art has been reported to have a positive influence on mood, its effectiveness in rehabilitation settings remains unclear.

Purpose:This study investigates the effects of rehabilitation and hospital art on mood improvement in patients admitted to a rehabilitation ward.

Methods:Thirty patients were assessed using questionnaires and semi-structured interviews at two time points, once before admission and then one week after hospitalization. Mood was measured using a visual analog scale, and interviews explored the perceived reasons behind mood changes.

Results: A significant improvement in mood was observed at the one-week mark following hospitalization (p=0.0389; 95% confidence interval: 0.51 to 18.22; Cohen's d=0.44). Rehabilitation was the most frequently cited factor contributing to improved mood. Some patients also referenced aspects of the care environment, including hospital art, while responding to the questionnaire. However, analysis of the interviews suggested a little association between rehabilitation and hospital art, indicating that hospital art had only a limited effect on mood improvement.

Conclusion: Rehabilitation emerged as the primary driver of mood improvement, although hospital art may have provided a limited supplementary effect. A positive treatment environment may enhance mood and support better functional outcomes.

## Introduction

Declining cognitive and physical functions associated with aging are significant contributors to the onset of depression among older adults. Depressive symptoms can result in reduced confidence levels in performing activities of daily living (ADL) [1]. Mood, whether positive or negative, has been shown to influence functional ability, with positive mood states being linked to better ADL performance and functional outcomes [2-4]. Therefore, mood is increasingly recognized as a critical factor in the rehabilitation process for older adults.

Although rehabilitation programs typically focus on physical recovery and functional improvement, environmental interventions such as hospital art, a technique that uses visual imagery to improve mood, have emerged as potential tools for promoting emotional well-being [5]. Hospital art, which involves the integration of visual artwork into care environments, has been reported to improve the mood of hospitalized cancer patients [6] and individuals undergoing surgery [7]. These findings suggest that the therapeutic environment plays a meaningful role in supporting patients’ emotional health.

However, within rehabilitation settings, the role of hospital art and similar environmental enhancements in mood improvement remains underexplored. While structured rehabilitation activities are known to have a positive influence on mood, the interaction between therapeutic interventions and the healing environment has not been fully investigated. Addressing this gap could lead to more holistic, patient-centered rehabilitation approaches.

Improving mood is particularly critical for older adults, as psychological readiness strongly influences motivation, participation in therapy, and overall functional recovery. Understanding whether hospital art can contribute to mood enhancement in rehabilitation settings may offer practical strategies for optimizing rehabilitation outcomes and improving the quality of care.

This preliminary study aimed to investigate the effects of rehabilitation and hospital art on mood improvement among patients admitted to a rehabilitation ward. This manuscript aims to address a gap in the literature regarding the role of environmental enhancements to emotional and functional recovery in the context of rehabilitation.

## Materials and methods

Participants

Patients who were consecutively admitted to the rehabilitation ward were recruited for this study. We excluded individuals who were deemed unable to answer the questionnaire or participate in the interview based on the judgment of physicians, physical therapists, or occupational therapists. A total of 30 participants were included in this study. The sample size was determined using a priori power analysis with G*Power version 3.1.9.7 (Heinrich-Heine-Universität Düsseldorf, Düsseldorf, Germany) [8], assuming a medium effect size, an alpha level of 0.05, and a power of 0.8. The calculation indicated that approximately 27 participants were required. Therefore, 30 participants were recruited, which was considered an appropriate sample size for this study.

Ethical considerations

The study protocol was reviewed and approved by the Ethics Committee of our institution (Approval No. 824). Participants provided both oral and written informed consent before enrollment. Information regarding hospital art was intentionally withheld during the consent process to minimize bias. The study was registered with a clinical trial registry (UMIN000055929).

Setting

The study was conducted in the rehabilitation ward of a hospital certified under Japanese institutional standards. Rehabilitation wards are designed for patients who require improvement in their ADL after completing treatment and provide individualized rehabilitation services without interruption, operating 365 days a year [9].

Intervention-rehabilitation and hospital art

Rehabilitation therapy was provided for up to 180 minutes per day, depending on each patient’s condition. In this study, patients received approximately 100 minutes of individualized rehabilitation therapy daily throughout their stay.

**Figure 1:**
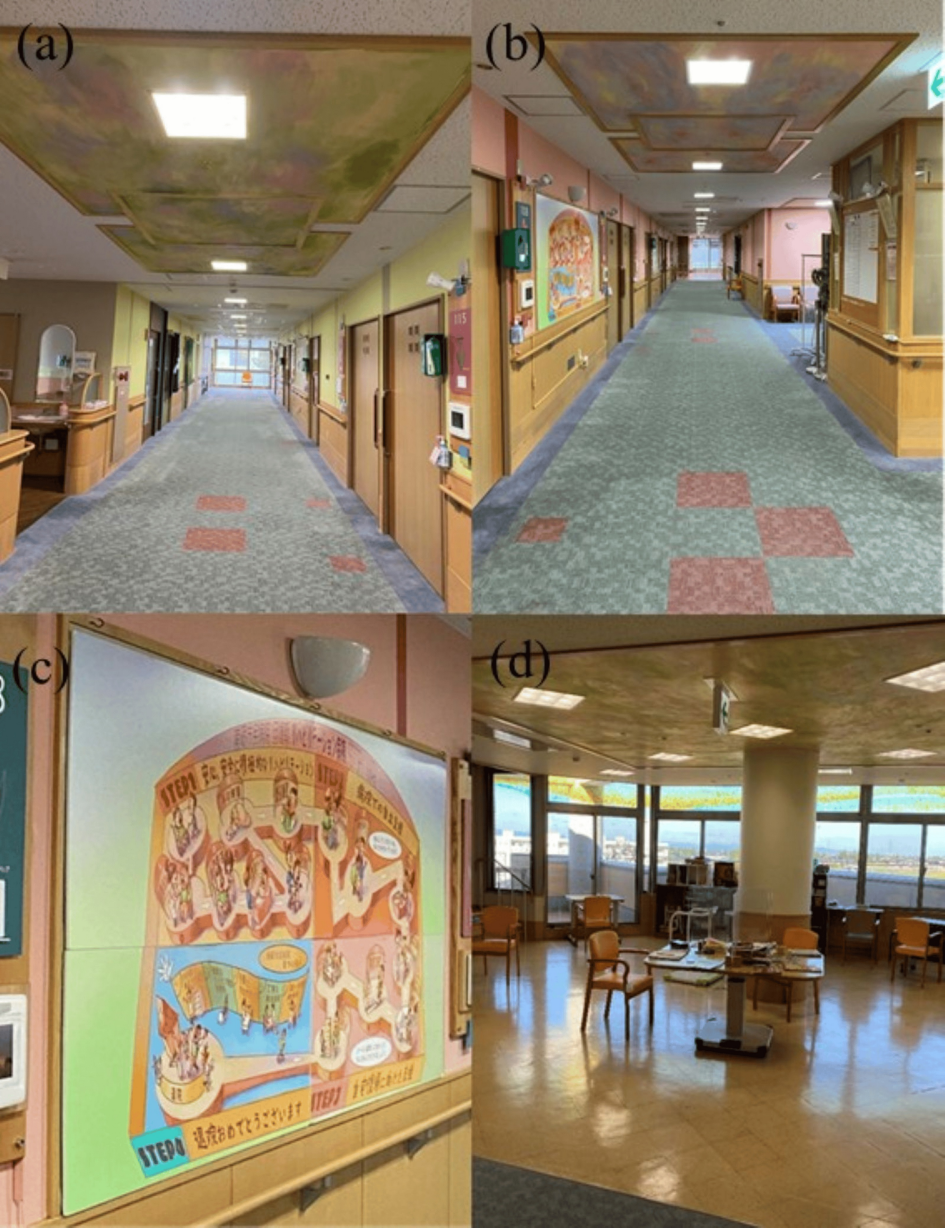
Hospital art (a) Green-centered artwork on the walls and ceilings of the corridors (b) Pink-centered artwork on the walls and ceilings of the corridors (c) An artwork portraying a patient with a positive outlook (d) Dayroom

The rehabilitation ward featured hospital art initiatives designed to foster a therapeutic environment (Figure 1). Each floor of the rehabilitation ward was decorated with hospital art. Specifically, the corridors’ walls and ceilings were adorned with green-themed artwork, designed to create a healing and reassuring atmosphere by evoking images of fresh greenery and rice paddies (Figure 1a). In another hallway, the walls and ceiling were decorated with pink-themed artwork. Pink tones were intended to relieve anxiety and reduce anger, thereby creating a calming environment (Figure 1b). Additionally, part of the hallway showcased paintings created by patients, depicting positive experiences from admission to discharge (Figure 1c). The dayroom, used both as a communal gathering space and a venue for functional training, featured both green and pink ceilings, and its windows were decorated with rainbow motifs, creating a bright and uplifting atmosphere (Figure 1d).

Design

This study employed a mixed-methods framework, incorporating both quantitative and qualitative approaches. Quantitative data were collected through mood assessments using a visual analog scale (VAS) at two time points: upon admission and after one week of hospitalization. The VAS is a 100 mm horizontal line on which 0 mm represents “feeling very poorly” and 100 mm represents “feeling very well”. The VAS was developed originally by the authors for this study. 

Data collection and procedures

Upon admission, patients completed the initial VAS assessment. A follow-up assessment was conducted after one week of hospitalization. If a change in mood was observed at the one-week mark, the reason for the change was explored through semi-structured interviews.

Qualitative data were collected through semi-structured interviews conducted after the second VAS assessment. Participants were asked, “Why did your mood change after being transferred to the ward?” All interviews were transcribed verbatim for qualitative analysis. To minimize potential bias, any information regarding the hospital art initiatives was intentionally withheld until data collection was complete. VAS and questions were developed originally by the authors for this study (Appendices).

Data analysis

Quantitative data are presented as mean ± standard deviation (SD). The statistical analysis of changes in VAS scores before and after hospitalization, normality was assessed using the Shapiro-Wilk test, confirmed that the data followed a normal distribution. Subsequently, paired t-tests were used to compare pre- and post-intervention scores. The p-value, 95% confidence interval (CI), and effect size (Cohen’s d) were calculated to evaluate the magnitude and significance of the change. Statistical significance was set at p<0.05. Analyses were performed using EZR version 1.68 (Saitama Medical Center; Jichi Medical University, Saitama, Japan) [10].

Qualitative data were analyzed using KH Coder software (Koichi Higuchi, Tokyo, Japan) [11]. Words extracted from the interview transcripts were subjected to hierarchical cluster analysis to identify common themes influencing mood changes.

## Results

Participant demographics

A total of 30 patients participated in the study (Table 1). The mean (± SD) age was 82.6±10.1 years, and 46.7% were male. The sample comprised 11 patients admitted following a stroke, 14 patients with orthopedic conditions, 4 patients with medical illnesses, and 1 patient after surgery.

**Table 1:** Participants' demographics (n=30)

Characteristic	Value
Age (mean±SD）	82.6±10.1years
Sex	
ー Male	14 (46.7%)
ー Female	16 (53.3%)
Diagnosis category	
ー Stroke	11 (36.7%)
ー Orthopedic conditions	14 (46.7%)
ー Medical illnesses	4 (13.3%)
ー Post-surgical condition	1 (3.3%)

Mood scores as measured by VAS

**Figure 2:**
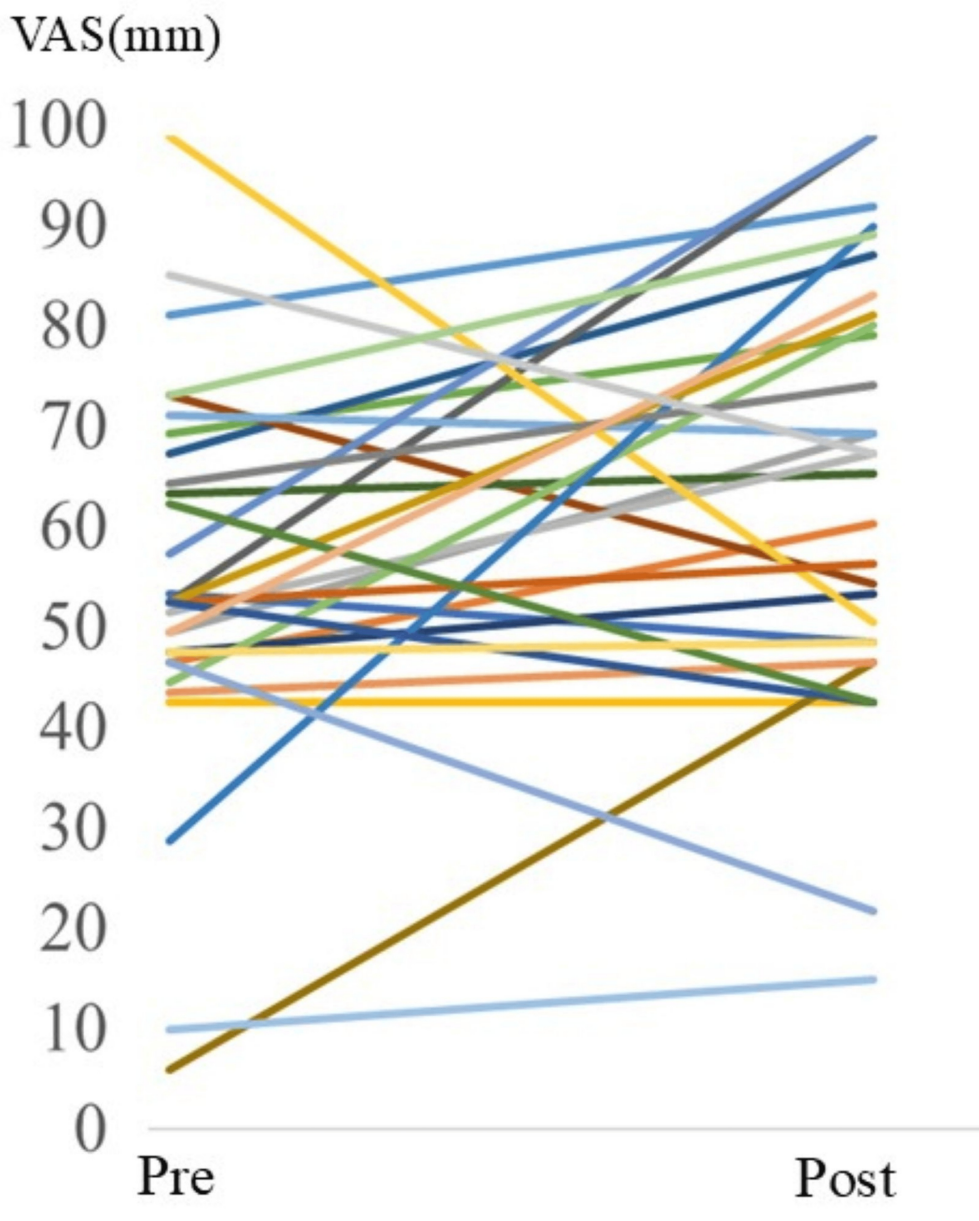
Mood assessment as measured by visual analog scale Pre: Admission Post: After one week of hospitalization

The mean (± SD) VAS score at admission was 55.4±20.1 mm. After one week of hospitalization, it increased to 64.7±21.8 mm (Figure 2). The difference in VAS scores (post-pre) was statistically significant (p=0.0389), with the 95% CI ranging from 0.51 to 18.22. The calculated effect size (Cohen’s d) was 0.44, indicating a small-to-moderate effect.

Interview results

Qualitative analysis of the semi-structured interviews revealed that “rehabilitation” was the most frequently mentioned word among the extracted terms. Human-related factors, including “people” and “staff,” were the next most frequently cited. Additionally, terms referring to the care environment, such as “ward,” “environment,” and “atmosphere,” also appeared commonly (Table 2).

**Table 2:** Extracted word list

Noun	Count	Frequency (%)
Rehabilitation	10	33.3
People	5	16.7
Staff	4	13.3
Environment	4	13.3
Ward	4	13.3
Motivation	3	10
Feeling	3	10
Mood	3	10
Atmosphere	3	10

**Figure 3:**
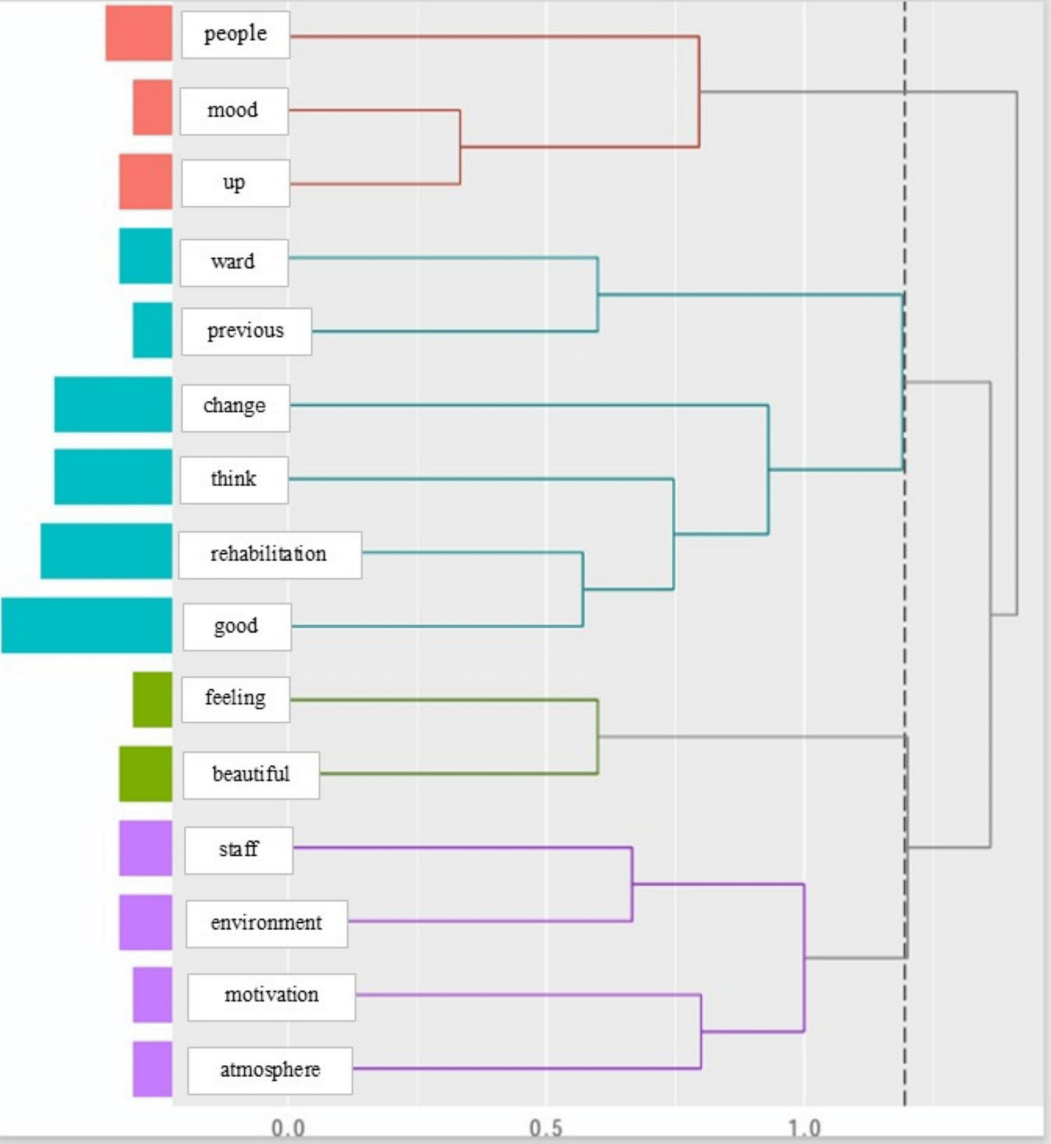
The hierarchical cluster

Hierarchical cluster analysis grouped the extracted words into three thematic groups, namely, category i: people, mood, up; category ii: ward, previous, change, think, rehabilitation, good; and category iii: feeling, beautiful, staff, environment, motivation, atmosphere (Figure 3).

## Discussion

Patients admitted to the rehabilitation ward demonstrated a statistically significant improvement in mood between admission and one week later. When asked about factors contributing to their improved mood, “rehabilitation” was the most frequently mentioned, followed by references to human interactions and then to aspects of the care environment.

Rehabilitation emerged as the primary factor associated with mood improvement. This finding is consistent with previous research suggesting that exercise therapy, a core component of rehabilitation, can enhance mood by stimulating the release of neurotransmitters [12] and improving psychological well-being [13]. The results also support the claim that rehabilitation interventions improve not only physical function but also contribute to emotional health.

Regarding the care environment (including hospital art), some patients referenced aesthetic elements with expressions such as “beautiful,” “atmosphere,” and “environment.” Although the phrase “hospital art” was not explicitly used, likely due to the older age of the study population, these comments appear to reflect perceptions of the physical environment, including the presence of hospital art. Prior studies have reported that hospital art, particularly nature-themed or abstract artwork, can reduce anxiety and physiological stress indicators in critically ill patients, such as those in ICUs or oncology wards [6,7]. These studies primarily focused on patients with high acuity and limited mobility. In contrast, the present study extends these findings to a population of individuals undergoing rehabilitation, who were generally more mobile and less severely ill. While the observed effect of the care environment on mood was modest, it suggests that environmental aesthetics may support mood even in lower-acuity settings.

However, results from the hierarchical cluster analysis indicated that references to rehabilitation and the care environment formed separate clusters, suggesting a weak association between the two. This separation implies that patients whose mood improves primarily through rehabilitation may not derive additional benefit from hospital art. These findings suggest that although rehabilitation serves as a strong mood enhancer for many individuals, environmental factors such as hospital art may serve as a modest adjunct in certain patients who may not experience sufficient mood improvement through rehabilitation.

Although the present study did not directly measure changes in ADL performance, previous research has reported an association between mood improvements and gains in ADL performance [2]. Achieving independence in ADL is a central goal of rehabilitation, and addressing both physical and psychological factors (including mood and motivation) is essential. The findings of this study suggest that fostering a positive emotional environment through both therapeutic interventions (such as rehabilitation) and environmental enhancements to the care setting may indirectly support the achievement of functional recovery goals.

Limitations

This study has several limitations. First, as a preliminary study, the sample size was small and lacked a control group, which limits the generalizability of the findings. Second, mood was assessed only at a single time point for follow-up (one week after admission); thus, it remains unclear whether the recorded mood improvements were sustained through the entire hospital stay. Third, the study did not address whether mood improvements translated into measurable gains in ADL.

In the future, we plan to increase the sample size and conduct a medium- to long-term intervention with a control group to clarify the effects of mood improvement on ADL outcomes and assess the long-term impact of environmental enhancements in rehabilitation settings.

## Conclusions

This study demonstrated that both rehabilitation and the care environment, including hospital art, independently contributed to mood improvement among patients admitted to a rehabilitation ward, with rehabilitation having the greatest impact. Although rehabilitation interventions were the primary drivers of mood enhancement, hospital art had a limited but independent supplementary effect on some patients. Given that improved mood has been associated with better ADL outcomes in prior research, these findings indicate that integrating therapeutic and environmental interventions could enhance the advancement of broader rehabilitation goals.

## Figures and Tables

**Figure 4:**
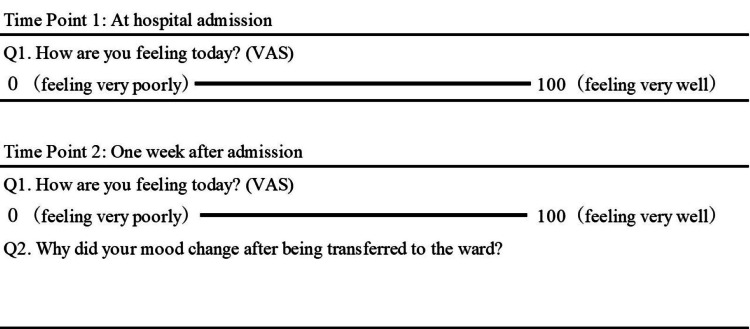
English translation of the questionnaire The following questions were developed originally by the authors for this study. Participants responded to the visual analog scale at two time points. At the second time point (one week after admission), an open-ended interview was also conducted.
